# Computational Characterization of β-Li_3_PS_4_ Solid Electrolyte: From Bulk and Surfaces to Nanocrystals

**DOI:** 10.3390/nano12162795

**Published:** 2022-08-15

**Authors:** Naiara Leticia Marana, Mauro Francesco Sgroi, Lorenzo Maschio, Anna Maria Ferrari, Maddalena D’Amore, Silvia Casassa

**Affiliations:** 1 Theoretical Group of Chemistry, Chemistry Department, Torino University, 10124 Torino, Italy; 2 Centro Ricerche Fiat S.C.p.A., Strada Torino 50, 10043 Orbassano, Italy

**Keywords:** solid-state electrolyte, lithium batteries, DFT, Li_3_PS_4_, Wulff nanocrystals

## Abstract

The all-solid-state lithium-ion battery is a new class of batteries being developed following today’s demand for renewable energy storage, especially for electric cars. The key component of such batteries is the solid-state electrolyte, a technology that promises increased safety and energy density with respect to the traditional liquid electrolytes. In this view, β-Li_3_PS_4_ is emerging as a good solid-state electrolyte candidate due to its stability and ionic conductivity. Despite the number of recent studies on this material, there is still much to understand about its atomic structure, and in particular its surface, a topic that becomes of key relevance for ionic diffusion and chemical stability in grain borders and contact with the other device components. In this study, we performed a density functional study of the structural and electronic properties of β-Li_3_PS_4_ surfaces. Starting from the bulk, we first verified that the thermodynamically stable structure featured slight distortion to the structure. Then, the surfaces were cut along different crystallographic planes and compared with each other. The (100) surface is confirmed as the most stable at *T* = 298 K, closely followed by (011), (010), and (210). Finally, from the computed surface energies, the Wulff nanocrystals were obtained and it was verified that the growth along the (100) and (011) directions reasonably reproduces the shape of the experimentally observed nanocrystal. With this study, we demonstrate that there are other surfaces besides (100) that are stable and can form interfaces with other components of the battery as well as facilitate the Li-migration according to their porous structures.

## 1. Introduction

In the last years, there has been an increase in the need for more efficient batteries, mainly driven by the transition towards electric car mobility and renewable energy sources. Research leads thus towards more efficient, portable batteries with a large energy storage capacity. Safety and reliability have become, more than ever, key aspects.

In this, all-solid-state-lithium batteries (ASSLBs) have shown good results because, in addition to improved safety and lifetime, they promise a good energy density, packaging, and operating temperature range. The main change concerning traditional batteries is the introduction of one (or eventually more) solid-state electrolyte (SSE), which main classes of SSE were recently reviewed in [1]. In general, ideal SSEs feature good ionic conductivity, high chemical stability, large electrochemical window, and good mechanical strength. Moreover, they should be environmentally benign and have low production costs. However, according to Homma et al. [2], a good SSE system should avoid non-metallic elements such as germanium and/or silicon. As a matter of fact, these elements might easily be reduced and oxidized during the electrochemical reaction, yielding higher electrochemical instability. This is an important condition for the solid electrolyte in solid-state batteries.

Among the materials studied as SSE, lithium thiophosphate, Li_3_PS_4_, stands out due to its good stability, atomic structure, ionic conductivity, and good chemical compatibility. At normal pressure and low temperature, Li_3_PS_4_ is formed as a γ-structure (space group *Pmn2_1_*), with hexagonal close-packed sulfide ion arrays in which the phosphorus ions are distributed over tetrahedral sites and the PS_4_ tetrahedrons are isolated from each other. With a slow cooling process from 900 °C and crystallizing to 520 °C [3], the γ is converted into a β-structure (space group *Pnma*), and by increasing the temperature, the phase transition occurs and the α is achieved (which belongs to a supergroup of *Pnma*) [4].

From the point of view of ionic conductivity, due to Li^+^ ions, the β-structure is the most interesting one with a value of 3.0 × 10^−2^ S cm^−1^ at 573 K (or 8.93 × 10^−7^ S cm^−1^ by extrapolating to room temperature) [2]. In 2013, Liu et al. [5] synthesized the nanoporous β-Li_3_PS_4_, which showed an ionic conductivity three orders of magnitude higher than that of the bulk (1.64 × 10^−4^ S cm^−1^ at room temperature). The ionic conductivity is favored by a fractional occupation of the sites, which does not occur in the γ structure, and which can explain the best conductivity in the β phase. Recently, Marchini et al. [6] revealed that the great conductivity of the nanoporous β-Li_3_PS_4_ is due to the portion of tetrahydrofuran (THF) that remains after the crystallization.

Although structural stability and good conductivity are important factors in having a good SSE and solid-state battery, good compatibility between the battery components is equally important, as interfacial resistance leads to a reduction in Li-ion transport. The interfacial resistance between the electrode active material and electrolyte is typically greater in all-solid-state lithium-ion batteries based on sulfide solid electrolytes due to the contact interface between the powders. Therefore, it is crucial to carry out interface engineering, i.e., to study and optimize the surface structure of each material that will compose the interface.

There are few studies dedicated to the characterization of Li_3_PS_4_ surfaces. However, to the best of our knowledge, a study on the different surfaces of the β-Li_3_PS_4_, their stabilities, and properties has not been carried out. For this reason, we have attempted detailed first-principle modeling of the β-Li_3_PS_4_ surfaces by computing their structural and electronic properties. According to the calculated surface energies, Wulff nanocrystal morphologies are proposed taking into account the increase or decrease of the stability of each surface.

## 2. Materials and Methods

All the calculations were performed with the CRYSTAL17 package [7], within the Density Functional Theory (DFT) method as developed for periodic systems in a basis set of localized Gaussian-type atomic functions, centered on each atom. Computational parameters were chosen and optimized by performing a preliminary analysis on the β-Li_3_PS_4_ bulk. In particular, the hybrid PBE0 functional [8,9] was adopted and the 6–11G [10], 86–311G* [11], and 85–211dG [12] basis sets were used to describe the Li, S, and P atoms, respectively.

The DFT integration was performed within a grid containing 99 radial and 1454 angular points, as specified by the XXLGRID keyword [7,13]. The accuracy of the truncation criteria for the bi-electronic integrals, Coulomb and HF exchange series, is controlled by a set of five thresholds for which the strict values of [8, 8, 8, 8, 16] were adopted. In the self-consistent field (SCF) procedure, the shrinking factor for both the diagonalization of the Fock matrix and the calculation of the energy was set to 4, corresponding to 4 independent k-points in the irreducible part of the Brillouin zone. The total and projected density of states (DOS) and the band structure were plotted using the same k-point sampling as in the SCF. The vibrational frequencies at the Γ point were computed within the harmonic approximation by diagonalizing the mass-weighted Hessian matrix [14,15].

Topological analysis of the electron density, *ρ*(r), was conducted according to the quantum theory of atoms in molecules and crystals (QTAIMC) as developed by Bader [16] and implemented in the CRYSTAL code [13,17], adopting the default values for all the computational parameters [18]. In the context of our investigation, it provided information on the bonding framework and helped highlight significant differences in the electronic structure of similar crystals. It consists of a few steps. First, the points where the gradient of the density vanishes, ∇*ρ*(r) = 0, i.e., the *ρ*(r) critical points, are found. Then, they are classified at local minima, maxima, and saddle points according to the sign of the second derivatives of the density [19]. Among these, the bond critical points (BCP) represent the saddle in the charge density, being a minimum along the atom–atom direction and a maximum in the two perpendicular directions. Interesting enough, chemical bonds can be classified according to the value of the Laplacian, ∇²*ρ*(r)*,* the potential energy density, V(r), the positive definite kinetic energy density, G(r), and the bond degree, H(r)/*ρ*(r), with H(r) = V(r) + G(r), as calculated in r = r_BCP_ [20]. Put simply, interactions can be subdivided in (i) *covalent*, if the Laplacian is negative and H(r) and V(r)/G(r) > 2, i.e., if there is an excess of potential energy at the BCP; (ii) *transit*, if the Laplacian is positive, the bond degree is close to zero and 1 < V(r)/G(r) < 2; (iii) *ionic and multipolar* (i.e., hydrogen bonds and van der Waals interactions) characterized by positive Laplacian and H(r) and V(r)/G(r) < 1 due to the dominance of kinetic energy at the BCP [21,22].

The β-Li_3_PS_4_ belong to the space group *Pnma*, with experimental cell parameters [2] *a* = 12.82 Å, *b* = 8.22 Å, and *c* = 6.12 Å. Starting from the fully relaxed bulk structure, surfaces were obtained by cutting along suitable crystallographic planes. According to the experimental powder diffraction pattern, the planes (221) and (210) have a higher intensity and these surfaces were generated in addition to the usual ones: (001), (100), (110), (010), (101), (011), and (111).

As the β-Li_3_PS_4_ bulk contains 4 units of Li_3_PS_4_ in the reference cell (32 atoms in total), the number of layers of each surface was chosen to be multiple of 4. Therefore, starting from the minimal 32-atom unit, slabs with an increasing number of layers were considered. For each surface, different terminations along the ±z-direction were analyzed in order to consider different types of low coordination atoms and surface morphologies (see Table S4). The results for each surface, considering the different thicknesses and possible terminations, are discussed in Topic S2 of the Supplementary Information (SI) and shown in Figures S1–S9.

The structural stability was determined by computing the surface energy (*E_surf_*), and formation energies (*E_form_*), calculated as $E_{surf}=\frac{\left(E_{slab}^{opt}-nE_{bulk}^{opt}\right)}{2\times A}$, and $E_{form}=\left(E_{bulk}^{opt}-E_{slab}^{ghost}\right)+\left(E_{slab}^{no-ghost}-E_{slab}^{opt}\right)$, where $E_{slab}^{opt}$ and $E_{bulk}^{opt}$ are the energy of the optimized surface (slab) and bulk, respectively, n is the number of bulk units in the slab, A is the surface area, $E_{slab}^{ghost}$ and $E_{slab}^{no-ghost}$ are the energies of the non-optimized surfaces with and without ghost functions. In *E_form_*, the basis set superposition error (BSSE) is incorporated, whose value is related to the atoms that belong to different structures approaching each other, interacting, and the overlap among their basis functions produces a not-genuine stabilization that is inversely proportional to the quality of the adopted basis set. In this work, a posteriori counterpoise (CP) method [23] was applied.

The thermodynamic stability was evaluated by computing the Gibbs free energy at *T* = 298 K, $G=E_{elec}+E_{0}+E_{vib}^{T}+PV-TS_{vib}$, where $E_{elec}$ is the electronic energy, $E_{0}$ the zero-point vibrational energy, $E_{vib}^{T}$ is the thermal contribution to the vibrational energy at *T* = 298 K, PV is the pressure versus volume, *T* = 298 K, and $S_{vib}$ is the vibrational entropy contribution.

## 3. Results

### 3.1. Bulk Analysis

As already mentioned, the performance of the β-Li_3_PS_4_ phase as an electrolyte is probably linked to a residual configurational disorder on the atomic scale. In the *Pnma* structure, the lithium atoms can occupy three distinct positions, corresponding to Wyckoff 8d, 4b, and 4c symmetry, for a total of 16 sites in which to place the 12 Li of the reference cell. At the macroscopic level, this translates into fractional occupation numbers, which experimentally, at finite temperature, proved to be 1.0 for 8d, 0.7 for 4b, and 0.3 for 4c. Since from the computational point of view it is necessary to maintain the symmetry of the space group, we have adopted the most probable configuration, occupying positions d and b, in line with most of the other theoretical studies [24,25].

First structure optimization of the *Pnma* crystal led to lattice parameters *a* = 12.96 Å, *b* = 8.08 Å, and *c* = 6.25 Å, in good agreement with experimental data [2], with a deviation of 1.09%, −1.07%, and 2.12%, respectively. However, in order to get rid of two imaginary frequencies, both related to the lithium atom in 4b, we released the symmetry constraints and performed a scan of geometries along the normal modes corresponding to the imaginary values [13]. The structure underwent structural deformation and turned into a *Pn2_1_a* crystal, a subgroup of *Pnma*, with lattice parameters *a* = 12.91 Å, *b* = 8.14 Å, and *c* = 6.23 Å, corresponding to a deviation of 0.70%, −0.97 %, and 1.80% from the experimental values of Homma et al. [2]. Its electron energy per cell was 0.09 eV lower than that of the *Pnma* system and the computed frequencies were then all positive. According to our results, in the *Pnma*, Li atoms have coordinates similar to those measured by Homma et al. [2] and computed by Yang and coworkers [24], while in the *Pn2_1_a* model they occupy 4a Wickoff positions, similar to those in the distorted B3C1 structure recently calculated by Lim et al. [26], see Table 1.

It is worth mentioning that the positions of P and S atoms are practically the same in the two models, and there are also other more than reasonable similarities given the substantial equivalence between the two structures.

From an analysis of the chemical bonding profile along the Li-S and P-S directions, using the QTAIMC indicators, we see that the bonding framework is practically the same in the *Pnma* and *Pn2_1_a* models, see Table 2. The four almost equivalent P-S bonds can be classified as covalent interactions based on the topological values in r = r_BCP_: i.e., a high charge density, the negative values of the Laplacian, and a V/G ratio greater than 3. The four Li-S bonds have a so-called transitory nature, sharing features with both the ionic (positive values of the Laplacian in r_BCP_) and covalent interactions (cylindrical shape of the charge density around the bond axes and directionality). The small differences found in the topological indicators are due to slight discrepancies in the first coordination spheres of Li atoms at different Wyckoff positions. Based on this analysis, it can be concluded that the Li-S interactions are weak and can be easily broken, leaving the lithium ion free to migrate within the porous material. This obviously plays an important part in Li diffusion.

The computed mechanical properties for both the *Pnma* and *Pn2_1_a* are close to literature data [24], see Table S2. In particular, the β-structure is considered a soft material and this is a property of great interest in the case of SSE devices as the volume of the various components varies during battery operation, and any rigidity would make the structure unstable. A close comparison shows that the *Pn2_1_a* presents Young and Bulk moduli greater than those of the *Pnma*, highlighting a slightly higher stiffness, but it remains an extremely deformable system.

Since the differences between structures are minimal and boil down to thermodynamic stability, in the following we will refer only to the *Pn2_1_a*, being a global minimum in the potential energy surface.

**Figure 1:**
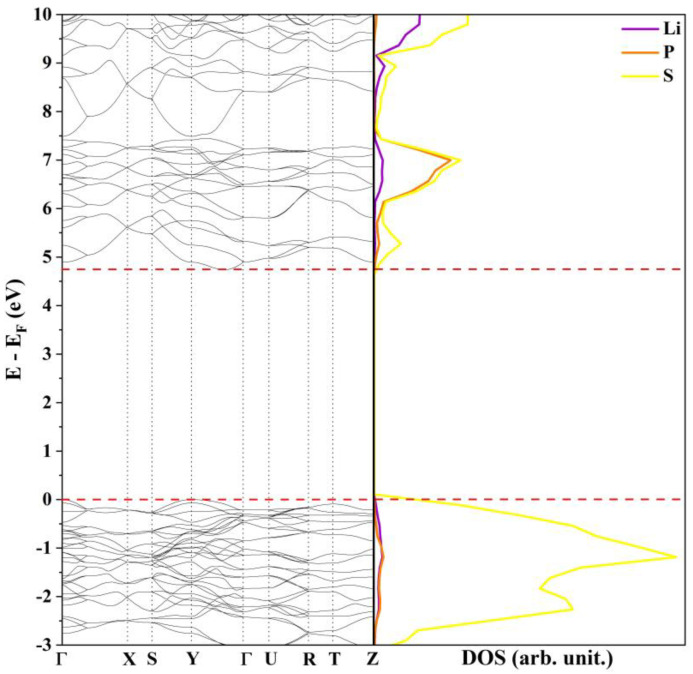
Band structure and density of states of β-Li_3_PS_4_ *Pn2_1_a* at the PBE0 level.

The Li_3_PS_4_ *Pn2_1_a* model is an insulator with an indirect band gap of 4.75 eV between the Γ and Z points. This value, computed at the PBE0 level, is in fairly good agreement with the one measured by Rangasamy et al. [27] of 5 eV and, as expected, is slightly overestimated when compared with those calculated in a previous theoretical study at the GGA-PBE and HSE06 [24] level. The band structure, reported in Figure 1, is very similar to those proposed by Lim and co-workers for the B3C1 and B4C0 systems [26]. From a qualitative point of view, the presence of wide, well-dispersed bands indicate small effective masses, band velocities, and large electron mobility. The projected density of states (DOS) shows that the low-energy electronic transitions occur between states that belong mainly to the *2p* orbitals of the sulfur atoms while Li and P provide an almost negligible contribution to the bands around the Fermi level.

**Figure 2:**
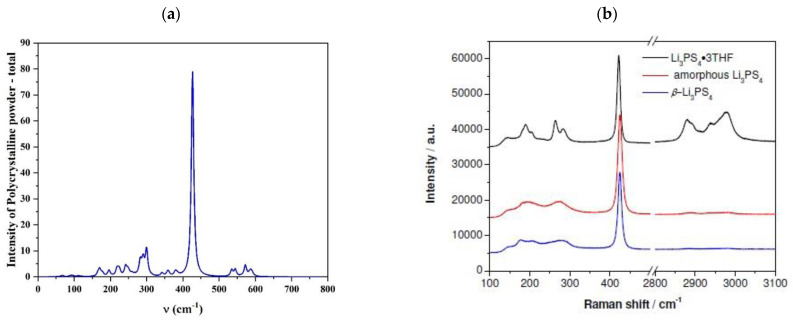
Raman spectra of β-Li_3_PS_4_ (**a**) *Pn2_1_a* crystal computed at the PBE0 level and (**b**) experimental obtained for different samples by Liu and co-workers [5].

Finally, to complete the characterization of the bulk material, we computed its Raman spectra and compared the results with experimental literature data [5,28]. The good agreement is encouraging and confirms the reliability of the adopted model for the bulk structure. The intense peak at 427 cm^−1^, Figure 2, represents the characteristic signal of the β-structure, measured at 422 cm^−1^ due to the collective vibrations of the (PS_4_) units. The wide band between 150 and 300 cm^−1^ contains the frequencies of all Li-S modes while weak signals above 500 cm^−1^ are assigned to the P-S bonds.

### 3.2. β-Li_3_PS_4_ Surfaces

Although unstable surfaces are more attractive for applications such as catalysis and photocatalysis, the materials involved in lithium-ion batteries must be stable due to the many changes and stresses they undergo during operation. In fact, there are different crystallographic planes along which the bulk phase can be reasonably cut, and each surface thus exposed can give rise to different terminations. For this reason, we have developed a strategy to identify the most stable surfaces, and only subsequently do we proceed to their characterization.

First, we noticed that the tetrahedron (PS_4_) represents an essential structural unit to maintain surface stability, so in modeling the various slabs we tried to preserve the integrity of these units as much as possible. Then, for each crystallographic plane, we designed slabs of increasing thickness, starting from the minimum reference cell, which contains—as in the bulk—four units of Li_3_PS_4_. We explored planes corresponding to the following Miller indices: (100), (010), (001), (110), (011), (111), (210), (211), according to the distribution density of the diffraction peaks as reported in Ref. [29], and, for each surface, different termination patterns, as LiS_3_, PS_3_, PS_2_, SLi_2_, SPLi_2_, LiS_2_, SPLi, and LiS, were modeled. All the results are collected and described in the Supplementary Information.

**Figure 3:**
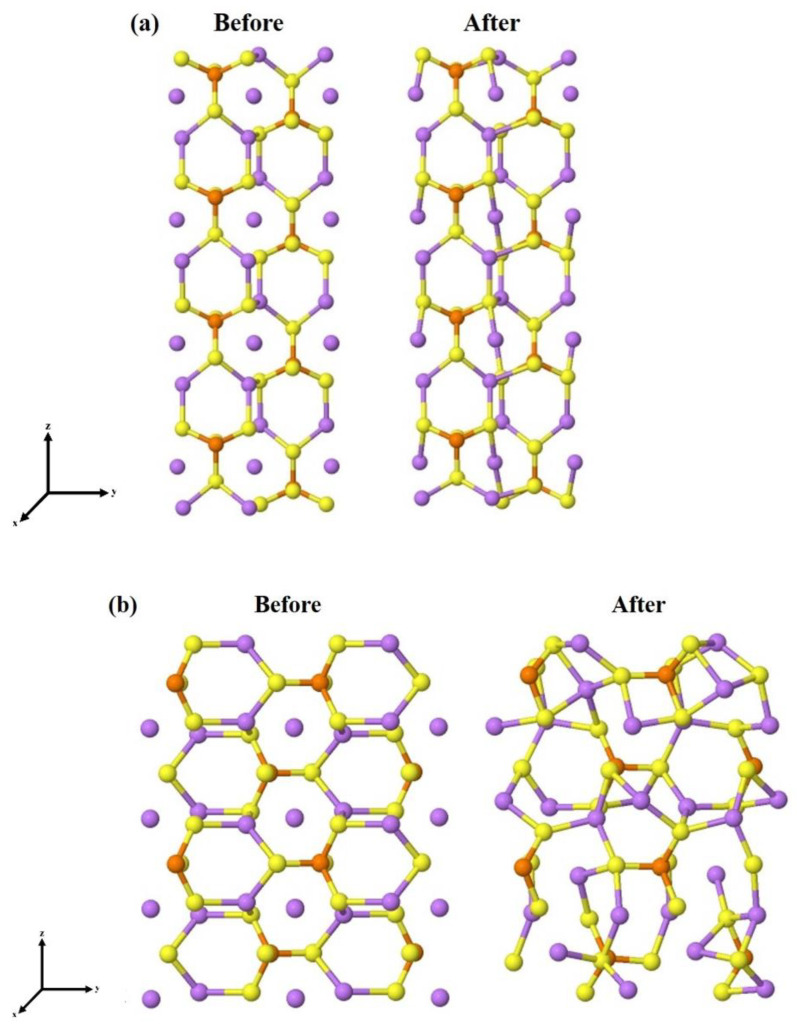

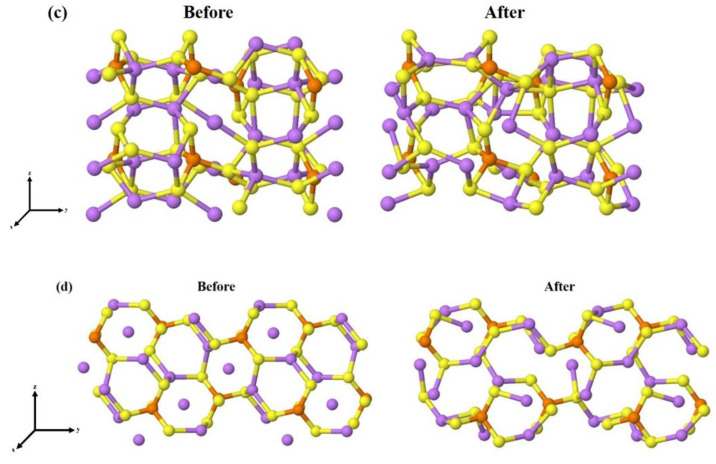
Stable surfaces cut from bulk, before and after geometry optimization (**a**) (100), (**b**) (010), (**c**) (011), and (**d**) (210). The spheres in purple, orange, yellow, and black are related to the lithium, phosphor, and sulfur atoms, respectively.

The complete optimization of the four unit slabs, i.e., atomic positions and lattice parameters, has led to a significant reduction in candidates because some surface models had undergone a drastic rearrangement and become particularly unstable. Moving on to the eight-unit structures (64 atoms each), we had already found the most stable surface terminations for each of the surfaces, so only these have been re-optimized. The structural and energetic information of the final models, containing eight units of Li_3_PS_4_, are reported in Table 3, while the initial and fully relaxed structures of the four most stable slabs, namely the (100), (010), (011), and (210), are shown in Figure 3.

In some cases, as for (001) and (110), the surface geometry after the optimization undergoes a significant reconstruction. In contrast, the LiS_2_-(100) surface, which is the most stable, experiences minimal distortion.

On the basis of *E_surf_*, see Table 3, it is possible to derive the following ranking: *(100) < (210) < (011) < (211) < (111) < (010) < (001) < (110)*. The correction due to the BSSE error produces a slightly different order, *(100) < (010) = (011) < (210) < (111) < (210) < (110) = (001)*, but the extremes remain the same: the surface (100) is the easiest to obtain, with a low *E_surf_* and *E_form_* close to zero, and the (110) surface is the most difficult, with high positive values of both *E_surf_* and *E_form_*. Then, the surface Gibbs free energy (*G*, expressed in kJ/(mol·m²)) is calculated for the four structures with the lowest surface energy, namely (100), (010), (011), and (210), and (100), *G* = 1.96 × 10^−19^, is confirmed as the most stable surface, followed by (210) (*G* = 2.11 × 10^−19^), (011) (*G* = 3.13 × 10^−19^), and (010) (*G* = 3.95 × 10^−19^).

**Figure 4:**
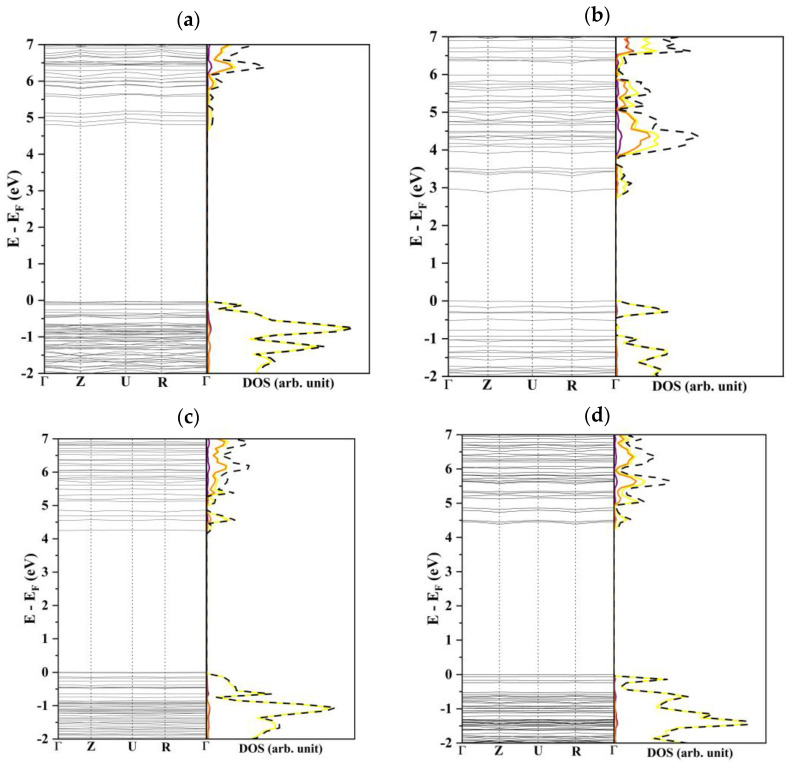
Band structure and density of states of (**a**) (100), (**b**) (010), (**c**) (011), and (**d**) (210). The curves in purple, orange, yellow, and black are related to the lithium, phosphor, sulfur, and total atoms contributions, respectively.

One interesting aspect to point out is that the four more stable slabs have very different electronic structures and properties. Their surface states, hereinafter referred to as band gap for uniformity of notation, vary from 2.7 eV for (010) to 4.6 in the case of (100). The DOS profiles of (100), (011), and (210), reported in Figure 4, are very similar: the valence bands are characterized by a strong contribution of the S-*2p* orbitals, only slightly hybridized with Li-*s* and P-*2p* functions while the conduction bands are mainly due to the P-*2p* orbitals. The (010) slab shows a rather peculiar density of states with a significant S character of the lower virtual bands and a consequent decrease in the band gap.

The Hirshfeld charges [30] were evaluated and compared with those of the bulk in Table 4 (and Table S4 for the less stable two-dimensional models). As expected, the charges of the atoms in the internal layers—which coordination is preserved and did not undergo structural rearrangements during the optimization—are very close to those in the bulk. In the case of the (100) slab, the surface atoms also show almost the same charge as those in the inner layers. On the contrary, the lithium and surface sulfur in (010) and (011) undergo a significant depletion of their atomic charge, as a consequence of the strong structural deformation.

**Figure 5:**
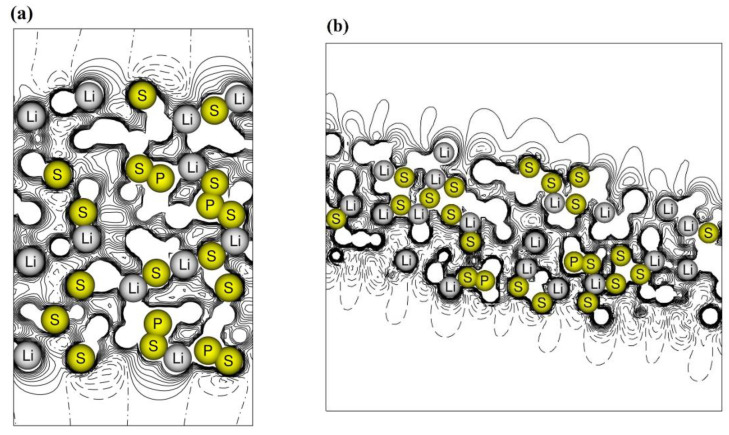
Electrostatic potential maps of the β-Li_3_PS_4_ surfaces (**a**) (100) and (**b**) (010). Continuous and dashed lines for positive and negative values of the potential.

The electrostatic potential maps along the z-direction are drawn in Figure 5 for the (100) and (010) surfaces. In the (100) slab, the potential is symmetric with respect to the central *xy* plane and has the same shape on the two surfaces. The net electric dipole along the non-periodic *z*-direction is zero and the film can grow, preserving stability. The situation is different in the case of slab (010), which exposes two different surfaces, one rich in lithium and the other dominated by sulfur atoms, see panel (b). A charge gradient along *z* is then established which increases with increasing thickness. On the one hand, the dipole opposes the growth of the film, but on the other hand, it can favor the migration of lithium ions along the structure.

To complete the analysis, the three-dimensional charge density of the two slabs was calculated and superimposed on the electrostatic potential to visualize the regions where the positive/negative potential is maximally concentrated, see Figure S10.

**Figure 6:**
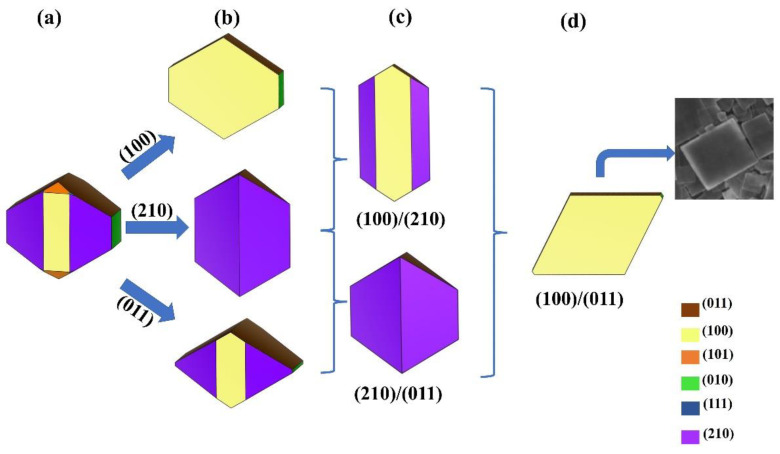
Different morphologies of β-Li_3_PS_4_ nanocrystals obtained by varying the relative stability of the various surfaces. Panel (**a**): the ideal nanocrystal based on our results. The blue arrows indicate the stabilization of the (100), (011), and (210) surfaces while the curly brackets indicate the simultaneous stabilization of the surfaces (100)/(011), (210)/(100), and (100)/(011). The resulting nanostructures are in the (**b**–**d**) panels, respectively. In the (**d**) panel, the SEM of β-Li_3_PS_4_ obtained by Hood and co-authors is also reported [31].

Finally, we used Wulff’s theory to visualize the ideal nanocrystal, which is the one that would form based on the calculated surface energies. Using the four most stable structures, we obtained the shape shown in Figure 6a. Then, it was possible to change the relative stability between different 2D models by gradually increasing a certain amount (~0.20 eV) of the surface energy of some surfaces with respect to others.

In this way, the growth of different surfaces can be favored or inhibited and the formation of different crystalline morphologies can be envisaged. In particular, in the ideal nanocrystal the (100), (011), and (210) surfaces dominate. By increasing the stability of one surface, we obtained the nanostructures as in Figure 6b. The structures in panel (c) emerge from the stabilization of two surfaces at the same time. Finally, in panel (d), the structure is represented when both the (100) and (011) surfaces are preferred: as can be seen from the inset, this morphology closely resembles that of the ultrathin nanoplates synthesized by Hood and co-authors [31]. These results show that reliable predictions are possible based on our calculations, i.e., the (100) and (011) films are among the most stable. Additionally, the different shapes for β-Li_3_PS_4_ nanocrystals are not only plausible but desirable due to the new properties they may have.

## 4. Conclusions

A DFT study of β-Li_3_PS_4_ bulk and surface stability was conducted using the CRYSTAL program, and by applying all-electron basis-sets and the PBE0 functional. This study mainly aims to investigate the stability of the 2D thin film of β-Li_3_PS_4_ for their use in lithium batteries as a solid electrolyte.

A full and symmetry-unconstrained geometry optimization of the experimental refined *Pnma* bulk has led to an equilibrium structure, belonging to the *Pn2_1_a* subgroup, in which the P-S lattice remains unchanged while the 12 Li atoms occupy three different Wyckoff positions: 4a′, 4a″, and 4a′′′. Then, a set of slabs, whose thickness varies between 9 to 23 Å, were modeled by cutting the bulk along different crystallographic directions. Based on the calculated surface formation energies and Gibbs free energies, the (100) surface resulted as the most stable, followed by (210), (011), and (010). In addition, the (100) and (210) films showed interesting features such as good mechanical and thermodynamic stability, a high concentration and good mobility of Li ions (i.e., the Li atoms are loosely bonded and the lattice is extremely porous), and a potentially small lattice mismatch with materials such as Li_2_S, widely used as a passivator in lithium technology. The (010) structure presents original characteristics, such as peculiar surface states and a net dipole moment along the non-periodic *z*-axis, which could eventually increase the diffusion rate of the lithium ions. Finally, Wulff’s theory was applied to derive the shapes of possible nanocrystals. As proof, it has been verified that the extra-stabilization of both the (100) and (011) surfaces, done by hand, leads to nanoplates very similar to those synthesized experimentally.

## Figures and Tables

**Table 1:** Lithium atomic coordinates on β-Li_3_PS_4_ for the *Pnma* and *Pn2_1_a* structures, compared with literature experimental and theoretical results.

Li-Sites	*Pnma*	*Pn2_1_a*	Experimental [2]	Theoretical [24]	Theoretical B3C1 [26]
**8d**	(0.332, 0.035, 0.388)	-	(0.356, 0.013, 0.439)	(0.333, 0.033, 0.394)	-
**4b**	(0.000, 0.000, 0.500)	-	(0.000, 0.000, 0.500)	(0.000, 0.000, 0.500)	-
**4a′**	-	(0.329, 0.043, 0.410)	-	-	(0.330, 0.041, 0.420)
**4a″**	-	(0.665, 0.972, 0.624)	-	-	(0.663, 0.973, 0.627)
**4a′′′**	-	(0.006, 0.045, 0.563)	-	-	(0.007, 0.053, 0.568)

**Table 2:** Value of several local quantities at the bond critical points in *Pn2_1_a* β-Li_3_PS_4_ structure: the bond distance (Å), the electron charge density (ρ(r)) (Å^−3^), the Laplacian of the density ($\nabla^{2}\rho$) (Å^−5^), the ratio between the potential energy density and the kinetic density (|V|/G), the bond degree (H/*ρ*) (a.u.) (i.e., the ratio between the total energy density and electron density), and the ellipticity *ε*. The indexes are related to the atom label.

	d	ρ(r)	$\nabla^{2}\rho$	|V|/G	H/ρ	ε
**Li_1_-S_30_**	2.502	0.0162	0.0847	0.8022	0.2156	0.0448
**Li_2_-S_17_**	2.456	0.0180	0.0933	0.8177	0.1990	0.0169
**Li_2_-S_28_**	2.448	0.0180	0.0911	0.8174	0.1945	0.0402
**Li_2_-S_18_**	2.459	0.0171	0.0875	0.8075	0.2060	0.0403
**Li_2_-S_29_**	2.483	0.0165	0.0862	0.8034	0.2140	0.0369
**Li_9_-S_17_**	2.445	0.0183	0.0925	0.8218	0.1911	0.0202
**Li_9_-S_21_**	2.526	0.0152	0.0761	0.8019	0.2066	0.0425
**Li_9_-S_32_**	2.612	0.0115	0.0596	0.7508	0.2570	0.0239
**Li_9_-S_28_**	2.618	0.0121	0.0615	0.7677	0.2382	0.1412
**Li_9_-S_25_**	2.713	0.0099	0.0494	0.7549	0.2440	0.3477
**P_16_-S_2_**	2.082	0.1369	−0.1844	3.297	−0.5960	0.0225
**P_15_-S_22_**	2.065	0.1411	−0.1942	3.286	−0.6110	0.0136
**P_13_-S_24_**	2.054	0.1433	−0.2003	3.266	−0.6210	0.0239
**P_13_-S_29_**	2.050	0.1439	−0.1976	3.2225	−0.6240	0.0327

**Table 3:** Surfaces’ termination (up/down), surface energy (*E_surf_*, in J/m²), formation energy (*E_form_*, in kJ/mol), and band gap (*E_gap_*, in eV) for the nine surfaces of β-Li_3_PS_4_ with eight units each.

	*Termination*	*E_surf_*	*E_form_*	*E_gap_*
**(001)**	LiS_2_/LiS_2_	2.15	31.94	1.82
**(100)**	LiS_2_/LiS_2_	0.91	2.19	4.66
**(010)**	LiS_2_/Li	1.83	5.16	2.70
**(101)**	PS_3_/SPLi	8.20	54.10	0.46
**(011)**	PS_3_/Li	1.43	5.24	4.24
**(110)**	SLi_2_/Li	2.27	32.22	1.99
**(111)**	LiS_3_/Li	1.60	10.91	2.21
**(210)**	SPLi/Li	0.99	5.88	4.20
**(211)**	LiS_2_/SPLi	1.57	11.84	2.14

**Table 4:** Hirshfield charges of β-Li_3_PS_4_ *Pn2_1_a* bulk and eight-unit surfaces for the surface (indicated by *) and internal atoms.

	Li_1_	Li*_1_	Li_2_	Li*_2_	P	P*	S_1_	S*_1_	S_2_	S*_2_	S_3_	S*_3_
**bulk**	1.007	1.007	1.011	1.011	1.573	1.573	−1.147	−1.147	−1.272	−1.272	−1.034	−1.034
**(001)**	1.006	1.004	1.008	0.993	1.580	1.127	−1.150	−1.023	−1.249	−1.206	−1.059	−1.176
**(100)**	1.007	1.003	1.010	1.008	1.575	1.498	−1.149	−1.130	−1.268	−1.297	−1.033	−1.020
**(010)**	1.006	0.979	1.008	1.011	1.611	1.500	−1.174	−0.945	−1.239	−1.253	−1.033	−1.035
**(011)**	1.009	1.001	1.013	1.012	1.566	1.588	−1.226	−0.974	−1.261	−1.022	−1.183	−0.836
**(210)**	1.009	0.972	1.011	1.002	1.565	1.549	−1.177	−1.006	−1.246	−1.180	−1.107	−1.001

## Data Availability

Not applicable.

## Supplementary Materials

The following supporting information can be downloaded at: https://www.mdpi.com/article/10.3390/nano12162795/s1, Table S1: Cell parameters and band gap of β-Li_3_PS_4_; Table S2: Mechanical properties of β-Li_3_PS_4_; Table S3: Possible termination along +z of each analyzed surface. The “X” represents the termination found in the surface and the ‘-’ the termination not found; Table S4: Hirshfield charges of β-Li_3_PS_4_ bulk and eight-unit surfaces for the surface (indicated by *) and internal atoms; Figure S1: (a) SLi_2_/SP and (b) LiS_2_/LiS_2_; Figure S2: (a) SLi_2_/SLi, (b) PS_3_/SP, (c) LiS_2_/LiS_2_, and (d) LiS/PS; Figure S3: (a) LiS_2_/Li and (b) PS_3_/SLi_2_; Figure S4: (a) PS_3_/LiS, (b) PS_3_/SPLi, (c) LiS_2_/PS_2_, and (d) LiS/LiS; Figure S5: (a) PS_3_/Li and (b) LiS_2_/Li; Figure S6: (a) SLi_2_/Li, (b) LiS/LiS, and (c) PS_2_/SPLi; Figure S7: (a) LiS_3_/LiS, (b) PS_2_/LiS_2_, (c) SLi_2_/LiS_2_, and (d) Li/PS_3_; Figure S8: (a) SPLi/Li, (b) LiS_2_/LiS, and (c) LiS_2_/SLi_2_; Figure S9: (a) SLi_2_/LiS, (b) LiS_2_/SPLi, and (c) PS_2_/LiS; Figure S10: Three-dimensional maps of the electronic charge density superimposed to the electrostatic potential of β-Li_3_PS_4_ surfaces along z-direction: (a) (100), (b) (210), and (c) (011). The spheres in purple, orange, yellow, and black are related to the lithium, phosphor, and sulfur atoms. The scale maps range from negative (−) in blue to positive (+) in red. References [2,24,25] were citied in the Supplementary Materials.

## References

[1] Ahniyaz A., de Meatza I., Kvasha A., Garcia-Calvo O., Ahmed I., Sgroi M.F., Giuliano M., Dotoli M., Dumitrescu M.A., Jahn M. (2021). Progress in Solid-State High Voltage Lithium-Ion Battery Electrolytes. Adv. Appl. Energy.

[2] Homma K., Yonemura M., Kobayashi T., Nagao M., Hirayama M., Kanno R. (2011). Crystal Structure and Phase Transitions of the Lithium Ionic Conductor Li_3_PS_4_. Solid State Ion..

[3] Mercier R., Malugani J.-P., Fahys B., Robert G., Douglade J. (1982). IUCr Structure Du Tetrathiophosphate de Lithium. Acta Crystallogr. Sect. B-Struct. Sci..

[4] Kim J.-S., Jung W.D., Choi S., Son J.-W., Kim B.-K., Lee J.-H., Kim H. (2018). Thermally Induced S-Sublattice Transition of Li_3_PS_4_ for Fast Lithium-Ion Conduction. J. Phys. Chem. Lett..

[5] Liu Z., Fu W., Payzant E.A., Yu X., Wu Z., Dudney N.J., Kiggans J., Hong K., Rondinone A.J., Liang C. (2013). Anomalous High Ionic Conductivity of Nanoporous β-Li_3_PS_4_. J. Am. Chem. Soc..

[6] Marchini F., Porcheron B., Rousse G., Blanquer L.A., Droguet L., Foix D., Koç T., Deschamps M., Tarascon J.M. (2021). The Hidden Side of Nanoporous β-Li_3_PS_4_ Solid Electrolyte. Adv. Energy Mater..

[7] Dovesi R., Erba A., Orlando R., Zicovich-Wilson C.M., Civalleri B., Maschio L., Rérat M., Casassa S., Baima J., Salustro S. (2018). Quantum-Mechanical Condensed Matter Simulations with CRYSTAL. Wiley Interdiscip. Rev. Comput. Mol. Sci..

[8] Perdew J.P., Wang Y. (1992). Accurate and Simple Analytic Representation of the Electron-Gas Correlation Energy. Phys. Rev. B.

[9] Adamo C., Barone V. (1999). Toward Reliable Density Functional Methods without Adjustable Parameters: The PBE0 Model. J. Chem. Phys..

[10] Dovesi R., Ermondi C., Ferrero E., Pisani C., Roetti C. (1984). Hartree-Fock Study of Lithium Hydride with the Use of a Polarizable Basis Set. Phys. Rev. B.

[11] Lichanot A., Aprà E., Dovesi R. (1993). Quantum Mechnical Hartree-Fock Study of the Elastic Properties of Li2S and Na2S. Phys. Status Solidi.

[12] Zicovich-Wilson C.M., Bert A., Roetti C., Dovesi R., Saunders V.R. (2001). Characterization of the Electronic Structure of Crystalline Compounds through Their Localized Wannier Functions. J. Chem. Phys..

[13] Dovesi R., Saunders V., Roetti C., Orlando R., Zicovich-Wilson C.M., Pascale F., Civalleri B., Doll K., Harrison N., Bush I. (2018). CRYSTAL17 User’s Manual. https://www.crystal.unito.it/manuals/crystal17.pdf.

[14] Pascale F., Zicovich-Wilson C.M., Gejo F.L., Civalleri B., Orlando R., Dovesi R. (2004). The Calculation of the Vibrational Frequencies of Crystalline Compounds and Its Implementation in the CRYSTAL Code. J. Comput. Chem..

[15] Zicovich-Wilson C.M., Pascale F., Roetti C., Saunders V.R., Orlando R., Dovesi R. (2004). Calculation of the Vibration Frequencies of α-Quartz: The Effect of Hamiltonian and Basis Set. J. Comput. Chem..

[16] Bader R.F.W. (1990). Atoms in Molecules: A Quantum Theory.

[17] Casassa S., Erba A., Baima J., Orlando R. (2015). Electron Density Analysis of Large (Molecular and Periodic) Systems: A Parallel Implementation. J. Comput. Chem..

[18] Gatti C., Casassa S. (2017). TOPOND14 User’s Manual. https://www.crystal.unito.it/topond/topond.pdf.

[19] Gatti C., Saunders V.R., Roetti C. (1994). Crystal Field Effects on the Topological Properties of the Electron Density in Molecular Crystals: The Case of Urea. J. Chem. Phys..

[20] Gatti C. (2005). Chemical Bonding in Crystals: New Directions. Z. Krist..

[21] Gatti C. (2013). Challenging Chemical Concepts through Charge Density of Molecules and Crystals. Phys. Scr..

[22] Clark A.E., Sonnenberg J.L., Hay P.J., Martin R.L. (2004). Density and Wave Function Analysis of Actinide Complexes: What Can Fuzzy Atom, Atoms-in-Molecules, Mulliken, Löwdin, and Natural Population Analysis Tell Us?. J. Chem. Phys..

[23] van Duijneveldt F.B., van Duijneveldt-van de Rijdt J.G.C.M., van Lenthe J.H. (1994). State of the Art in Counterpoise Theory. Chem. Rev..

[24] Yang Y., Wu Q., Cui Y., Chen Y., Shi S., Wang R.Z., Yan H. (2016). Elastic Properties, Defect Thermodynamics, Electrochemical Window, Phase Stability, and Li^+^ Mobility of Li_3_PS_4_: Insights from First-Principles Calculations. ACS Appl. Mater. Interfaces.

[25] Lepley N.D., Holzwarth N.A.W., Du Y.A. (2013). Structures, Li+ Mobilities, and Interfacial Properties of Solid Electrolytes Li_3_PS_4_ and Li3PO4 from First Principles. Phys. Rev. B.

[26] Lim M.S., Jhi S.H. (2018). First-Principles Study of Lithium-Ion Diffusion in β-Li_3_PS_4_ for Solid-State Electrolytes. Curr. Appl. Phys..

[27] Rangasamy E., Li J., Sahu G., Dudney N., Liang C. (2014). Pushing the Theoretical Limit of Li-CFx Batteries: A Tale of Bifunctional Electrolyte. J. Am. Chem. Soc..

[28] Ates T., Neumann A., Danner T., Latz A., Zarrabeitia M., Stepien D., Varzi A., Passerini S., Ates T., Neumann A. (2022). Elucidating the Role of Microstructure in Thiophosphate Electrolytes—A Combined Experimental and Theoretical Study of β-Li_3_PS_4_. Adv. Sci..

[29] Wang H., Hood Z.D., Xia Y., Liang C. (2016). Fabrication of Ultrathin Solid Electrolyte Membranes of β-Li_3_PS_4_ Nanoflakes by Evaporation-Induced Self-Assembly for All-Solid-State Batteries. J. Mater. Chem. A.

[30] Bultinck P., Van Alsenoy C., Ayers P.W., Carbó-Dorca R. (2007). Critical Analysis and Extension of the Hirshfeld Atoms in Molecules. J. Chem. Phys..

[31] Hood Z.D., Wang H., Pandian A.S., Peng R., Gilroy K.D., Chi M., Liang C., Xia Y. (2018). Fabrication of Sub-Micrometer-Thick Solid Electrolyte Membranes of β-Li_3_PS_4_ via Tiled Assembly of Nanoscale, Plate-Like Building Blocks. Adv. Energy Mater..

