# Supplementary material for: Computational Characterization of β-Li3PS4 Solid Electrolyte: From Bulk and Surfaces to Nanocrystals
Source: Nanomaterials (Basel). 2022 Aug 15;12(16):2795. doi: 10.3390/nano12162795 (PMC9416343; doi:10.3390/nano12162795)
Supplement: Supplementary file 1 [file nanomaterials-12-02795-s001.zip › nanomaterials-1841450-SI.pdf]

Supplementary Materials

Computational Characterization of β-Li_3PS_4 Solid Electrolyte: From Bulk and Surfaces to Nanocrystals

Naiara Leticia Marana [1,*], Mauro Francesco Sgroi [2], Lorenzo Maschio [1], Anna Maria Ferrari [1], Maddalena D’Amore [1] and Silvia Casassa [1,*]

[1] Theoretical Group of Chemistry, Chemistry Department, Torino University, 10124 Torino, Italy
[2] Centro Ricerche Fiat S.C.p.A., Strada Torino 50, 10043 Orbassano, Italy
* Correspondence: naiaraleticia.marana@unito.it (N.L.M.); silvia.casassa@unito.it (S.C.)

Topic S1: Bulk data

Table S1: Cell parameters and band gap of β-Li_3PS_4

**β-Li_3PS_4 (Pnma)**	*a* (Å)	*b* (Å)	*c* (Å)	E_{gap} (eV)
**Experimental**[1]	12.82	8.22	6.12	5.0
**Theoretical**[2]	12.86	7.76	6.21	2.2 – 3.7
**PBE**	13.03	8.13	6.29	2.70
**PBE0**	12.96	8.08	6.25	4.71
**HSE06**	12.96	8.09	6.25	3.97

Table S2: Mechanical properties of β-Li_3PS_4

	**Bulk modulus**	**Young's modulus**	**Poisson ratio**	c_{11}	c_{12}	c_{13}	c_{22}	c_{23}	c_{33}	c_{44}	c_{55}	c_{66}
**Theooretical.**[3]	21.39	28.90	0.27	47.82	15.93	17.90	36.23	10.20	28.46	12.88	9.81	12.17
Pnma	21.82	32.35	0.26	53.33	16.46	18.24	38.38	10.48	31.31	13.72	11.66	13.64
$Pn2_1a$	24.31	33.58	0.27	51.57	18.94	18.66	36.52	13.63	37.96	15.69	11.71	14.24

Topic S2: β-Li_3PS_4 surfaces

Table S3: Possible termination along +z of each analyzed surface. The "X" represents the termination found in the surface and the '-' the termination not found

	**LiS_3**	**PS_3**	**PS_2**	**SLi_2**	**S**	**Li**	**$SPLi_2$**	**LiS_2**	**SPLi**	**LiS**
**(100)**	-	X	-	X	-	X	-	X	-	-
**(001)**	X	X	-	-	-	-	X	X	-	X
**(010)**	-	X	-	-	-	X	-	X	-	-
**(101)**	-	X	-	X	X	-	X	X	-	-
**(011)**	-	X	X	-	-	-	X	X	X	X
**(110)**	X	-	X	X	-	-	-	X	-	X
**(111)**	X	X	X	X	-	-	-	-	X	X
**(210)**	-	X	X	-	-	-	-	X	X	-
**(211)**	X	-	X	X	-	-	X	X	-	-

Topic S3: Discussion on the choice of surface terminations

- **(001)**

In order to maintain the 4 units in the surface as in the bulk, the analyzed surfaces (001) contain 22 layers each. According to our analysis, only 2 terminations were judged to be possible candidates for the study. The surfaces are shown below. As the termination LiS_2/LiS_2 (not symmetric) presented the minor E_{surf}, it was calculated with 8 units of Li_3PS_4.

(a) $E_{surf} = 5.15$ J/m² (b) $E_{surf} = 1.89$ J/m²

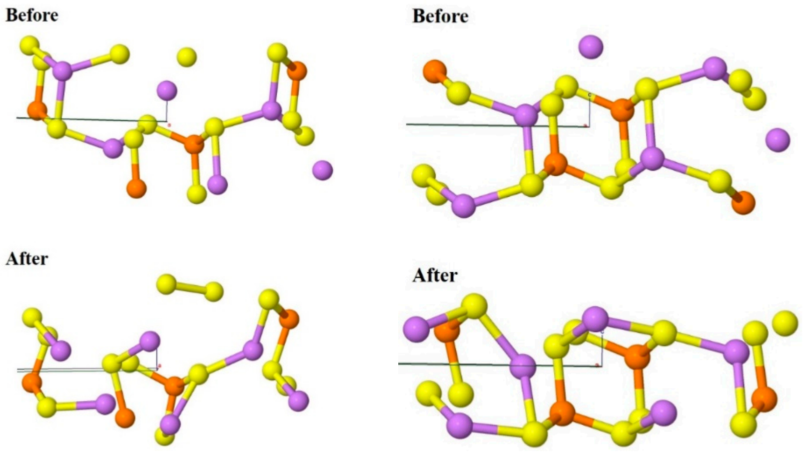

Figure S1: (a) SLi_2/SP and (b) LiS_2/LiS_2

- **(100)**

For this surface, also 22 layers correspond to 4 units of Li_3PS_4. Four terminations were analyzed (see below), as the termination denominated LiS_2/LiS_2 presented the minor E_{surf}, the surface with 8 units was calculated and the E_{surf} obtained is 0.91 J/m², i. e., 0.90 J/m² minor than the surface with 4 units.

(a) E_{surf} = 7.79 J/m² (b) E_{surf} = 5.82 J/m² (c) E_{surf} = 1.80 J/m² (d) E_{surf} = 5.35 J/m²

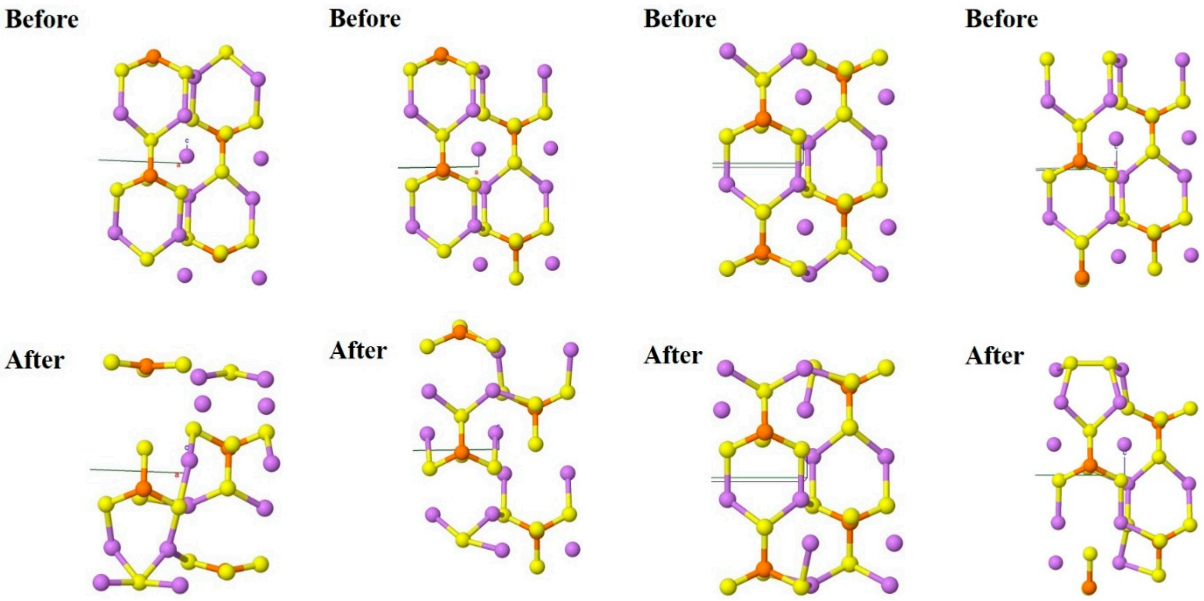

Figure S2: (a) SLi_2/SLi, (b) PS_3/SP, (c) LiS_2/LiS_2, and (d) LiS/PS

- **(010)**

Eight layers of this surface have 4 units of Li_3PS_4. This surface has only two possible terminations: LiS_2/Li and PS_3/SLi_2 (see below). The surface with termination LiS_2/Li presented the minor E_{surf} and it was calculated with 8 units of Li_3PS_4. After optimization, the E_{surf} of (010) with 8 units is 1.83 J/m² and decreases 1.20 J/m² from the E_{surf} of (010) with 4 units.

(a) E_{surf} = 3.03 J/m² (b) E_{surf} = 4.25 J/m²

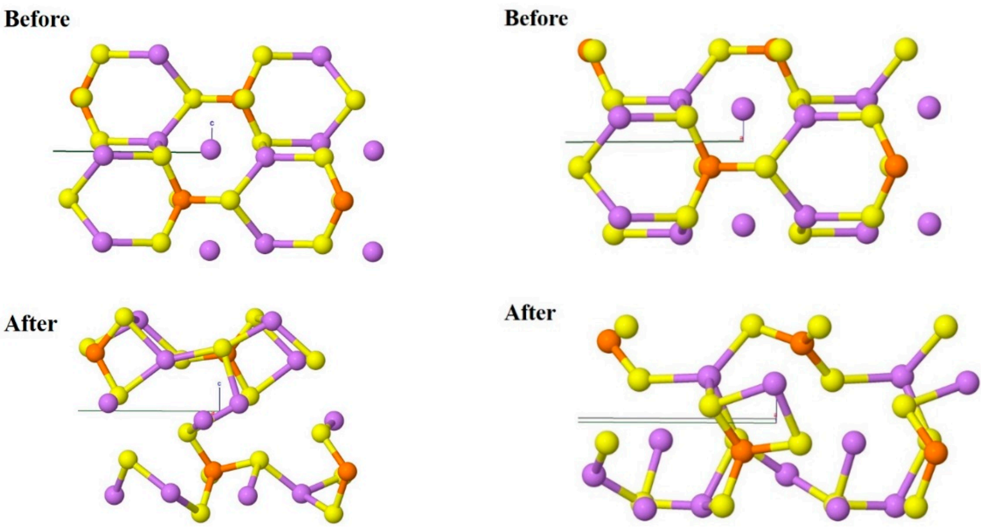

Figure S3: (a) LiS_2/Li and (b) PS_3/SLi_2

- **(101)**

The surface (101) was made with 21 layers to respect the 4 units of Li_3PS_4. Only the 4 terminations below were considered relevant. However, only the termination called LiS/LiS maintains the cluster [PS_4] integer, that is the surface with minor E_{surf}, 1.54 J/m² and it was calculated with 8 units of Li_3PS_4 which E_{surf} obtained was 8.20 J/m². Therefore, this termination's surface showed to be unstable.

(a) E_{surf}= 4.29 J/m² (b) E_{surf}= 2.65 J/m²

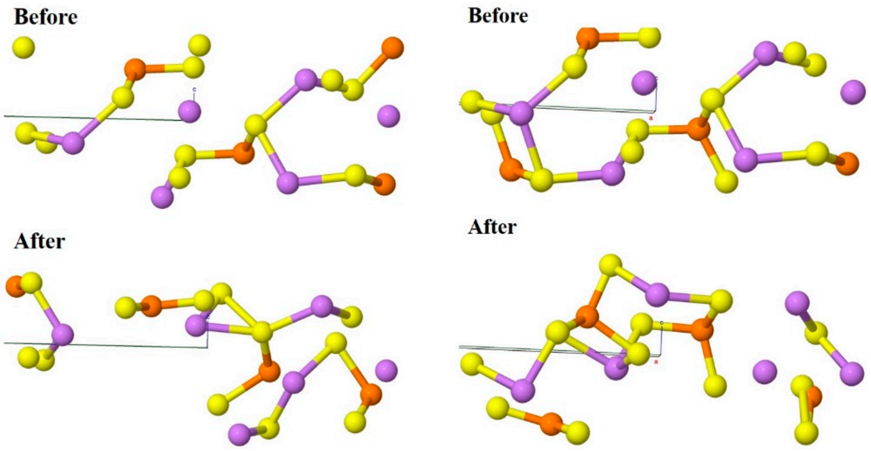

(c) E_{surf}= 3.70 J/m² (d) E_{surf}= 1.54 J/m²

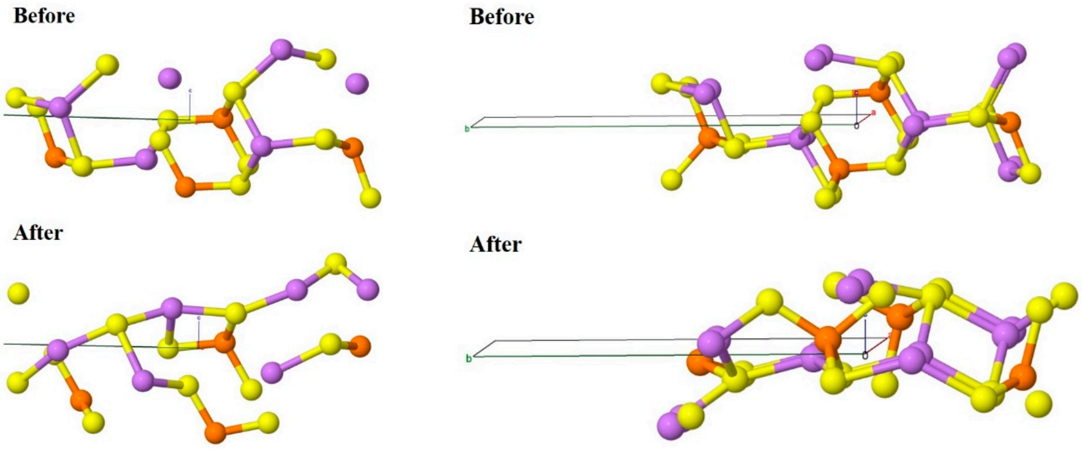

Figure S4: (a) PS_3/LiS, (b) PS_3/SPLi, (c) LiS_2/PS_2, and (d) LiS/LiS

- **(011)**

The 4 units of Li_3PS_4 are given by 16 layers of the (011) surface. Two terminations were considered relevant due to the many unbonded atoms in the other terminations (see below). The termination PS_3/Li presented the minor E_{surf} and it was calculated with 8 units of Li_3PS_4, which E_{surf} obtained was 1.50 J/m², i. e., no changes in the surface energy formation were observed by the increasing number of units.

(a) E_{surf}= 1.50 J/m² (b) E_{surf}= 2.77 J/m²

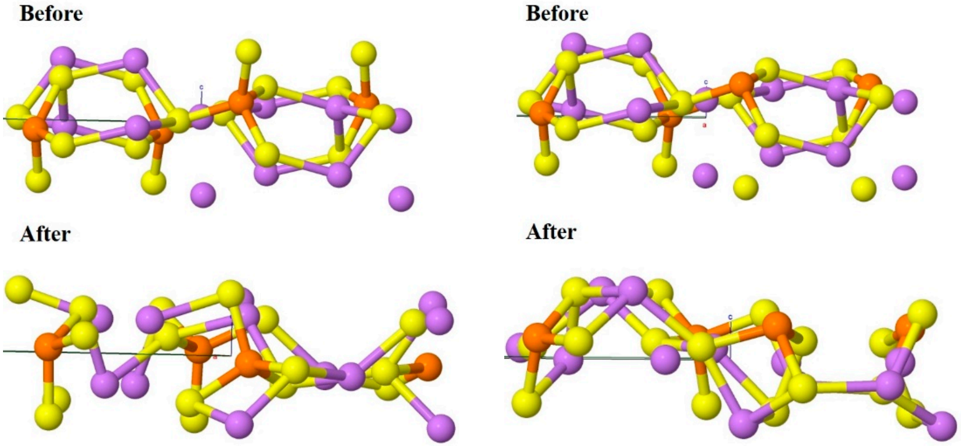

Figure S5: (a) PS_3/Li and (b) LiS_2/Li

- **(110)**

The (110) surface was initially studied with 4 units of Li_3PS_4, which is composed of 30 layers. Only 3 terminations were considered interesting for this study (see below). As the termination SLi_2/Li presented the minor E_{surf}, was calculated the surface with 8 units of Li_3PS_4, also, the termination LiS/LiS (considered symmetric) was calculated with 8 units. Both terminations with 8 units have, almost, the same E_{surf}, 2.24 and 2.27 J/m² for LiS/LiS and SLi_2/Li, respectively, a reduction of 40% and 30% concerning the 4 units models. However, the termination SLi_2/Li presented the smallest structural distortion after the optimization and kept the tetrahedron [PS_4].

(a) $E_{surf} = 3.22$ J/m²

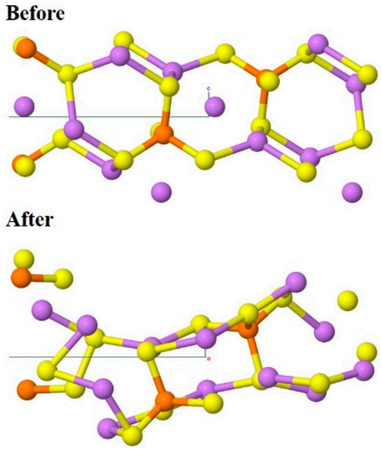

(b) $E_{surf} = 4.78$ J/m²

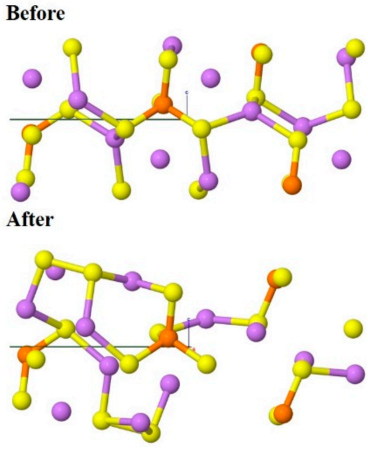

(c) $E_{surf} = 3.67$ J/m²

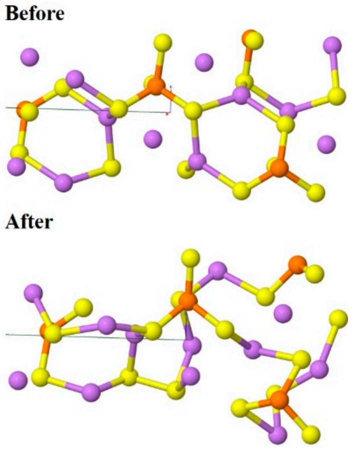

Figure S6: (a) SLi_2/Li, (b) LiS/LiS, and (c) PS_2/SPLi

- **(111)**

The surface (111) needs 30 layers to have 4 units of Li_3PS_4. No symmetric terminations were found for this surface. The terminations analyzed were LiS_3/LiS, PS_2/LiS_2, SLi_2/LiS_2, and Li/PS_3 (see below). The termination LiS_3/LiS presented the minor E_{surf} and it was calculated taking to account 8 units of Li_3PS_4. After full optimization, the E_{surf} is 1.60 J/m², i. e., a reduction of ~40%.

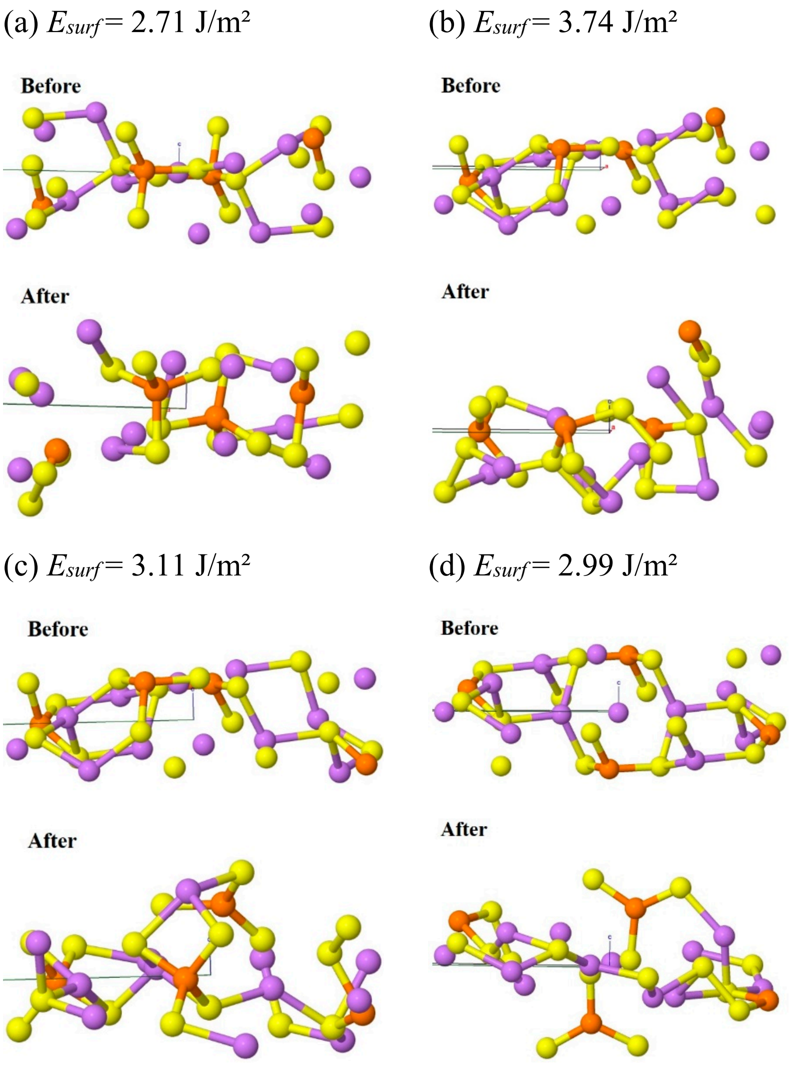

Figure S7: (a) LiS_3/LiS, (b) PS_2/LiS_2, (c) SLi_2/LiS_2, and (d) Li/PS_3

- **(210)**

The surface (210) with 16 layers is composed of 4 units of Li_3PS_4. It was seen that only 4 terminations are relevant for the present study (see below). The termination SPLi/Li presented the minor E_{surf} and it was optimized with 8 units. After optimization, it was found an E_{surf} = 0.99 J/m², a reduction of 41%.

(a) E_{surf}= 1.70 J/m²

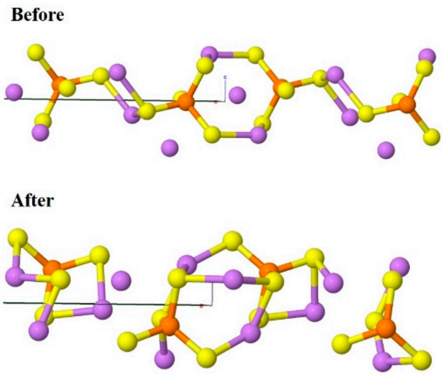

(b) E_{surf}= 3.18 J/m²

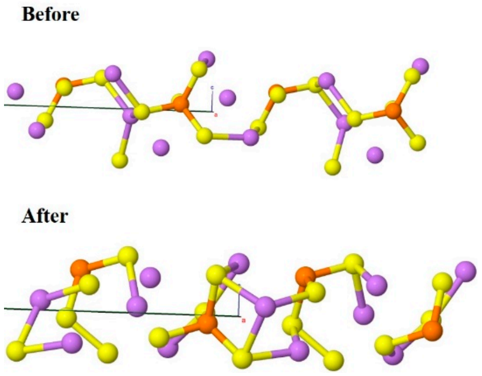

(c) E_{surf}= 3.06 J/m²

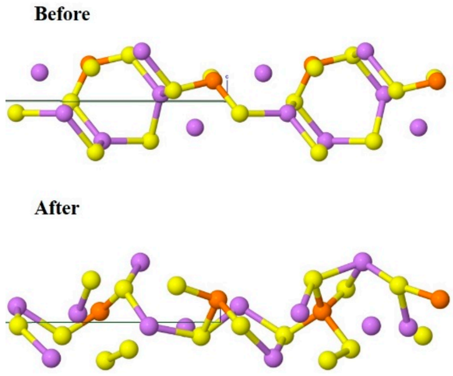

Figure S8: (a) SPLi/Li, (b) LiS_2/LiS, and (c) LiS_2/SLi_2

- **(211)**

The (211) surface was analyzed with 30 layers (4 units of Li_3PS_4). No symmetric terminations were found. The 3 relevant terminations are represented below. It was found that the termination LiS_2/SPLi presents the minor E_{surf}, and a model with 8 units was also calculated. The E_{surf} of 8 units is reduced ~51%, 1.57 J/m².

(a) E_{surf}= 3.62 J/m²

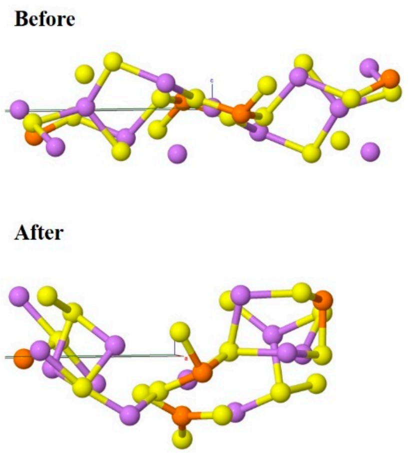

(b) E_{surf}= 3.04 J/m²

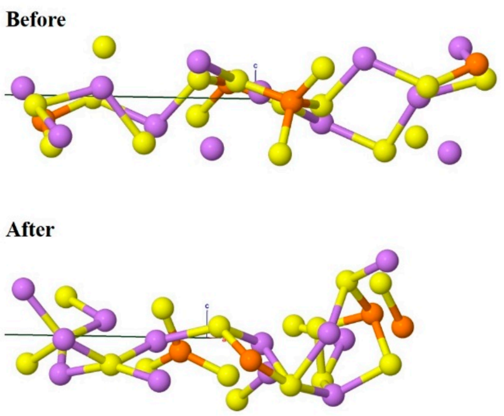

(c) E_{surf}= 4.27 J/m²

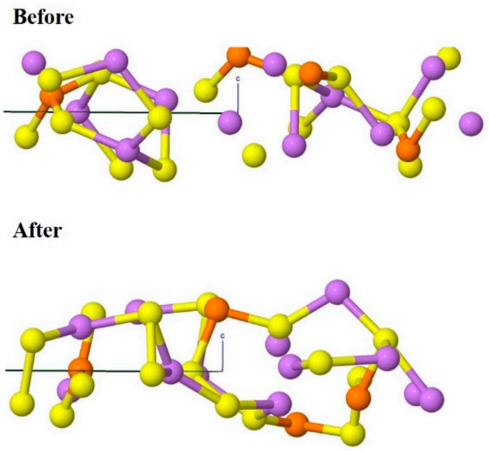

Figure S9: (a) SLi_2/LiS, (b) LiS_2/SPLi, and (c) PS_2/LiS

Table S4: Hirshfield charges of β-Li_3PS_4 bulk and 8-unit surfaces for the surface (indicated by *) and internal atoms.

	**Li_1**	**Li^*_1**	**Li_2**	**Li^*_2**	**P**	**P***	**S_1**	**S^*_1**	**S_2**	**S^*_2**	**S_3**	**S^*_3**
**bulk**	**1.007**	**1.007**	**1.011**	**1.011**	**1.573**	**1.573**	**-1.147**	**-1.147**	**-1.272**	**-1.272**	**-1.034**	**-1.034**
**(001)**	1.006	1.004	1.008	0.993	1.580	1.127	-1.150	-1.023	-1.249	-1.206	-1.059	-1.176
**(100)**	1.007	1.003	1.010	1.008	1.575	1.498	-1.149	-1.130	-1.268	-1.297	-1.033	-1.020
**(010)**	1.006	0.979	1.008	1.011	1.611	1.500	-1.174	-0.945	-1.239	-1.253	-1.033	-1.035
**(011)**	1.009	1.001	1.013	1.012	1.566	1.588	-1.226	-0.974	-1.261	-1.022	-1.183	-0.836
**(110)**	1.009	1.001	1.009	1.008	1.410	1.024	-1.161	-0.706	-1.273	-1.154	-1.187	-0.799
**(111)**	1.005	0.959	1.009	1.009	1.657	0.605	-1.158	-0.948	-1.304	-1.192	-1.078	-0.973
**(210)**	1.009	0.972	1.011	1.002	1.565	1.549	-1.177	-1.006	-1.246	-1.180	-1.107	-1.001
**(211)**	1.007	1.002	1.016	1.013	1.508	0.569	-1.130	-0.971	-1.229	-2.004	-1.098	-0.530

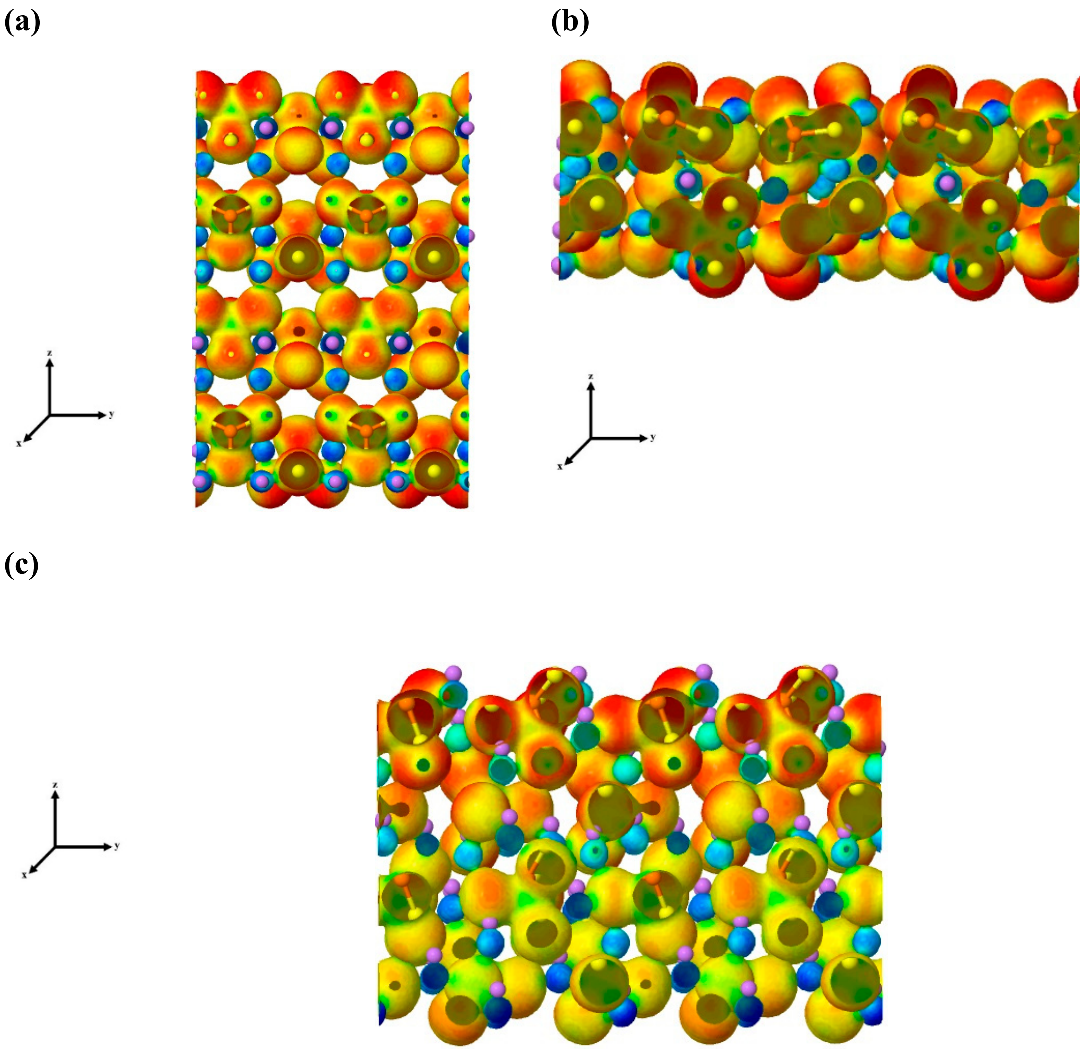

Figure S10: Three-dimensional maps of the electronic charge density superimposed to the electrostatic potential of β-Li_3PS_4 surfaces along z-direction: (a) (100), (b) (210), and (c) (011). The spheres in purple, orange, yellow, and black are related to the lithium, phosphor, and sulfur atoms. The scale maps range from negative (-) in blue to positive (+) in red.

References

1. Homma, K. *et al.* Crystal structure and phase transitions of the lithium ionic conductor Li3PS4. *Solid State Ionics* **182**, 53–58 (2011).

2. Lepley, N. D., Holzwarth, N. A. W. & Du, Y. A. Structures, Li+ Mobilities, and Interfacial Properties of Solid Electrolytes Li3PS4 and Li3PO4 from First Principles. *Phys. Rev. B* **88**, 104103 (2013).

3. Yang, Y. *et al.* Elastic Properties, Defect Thermodynamics, Electrochemical Window, Phase Stability, and Li+ Mobility of Li3PS4: Insights from First-Principles Calculations. *ACS Appl. Mater. Interfaces* **8**, 25229–25242 (2016).
